# A review of the application of digital phenotyping in predicting peripartum depressive symptoms

**DOI:** 10.1038/s41746-026-02653-y

**Published:** 2026-04-24

**Authors:** Boglarka Z. Kovacs, Sascha Schweitzer, Fotios C. Papadopoulos, Annette Bauer, Alkistis Skalkidou, Hsing-Fen Tu

**Affiliations:** 1 Erasmus University Medical Center, Erasmus MC, Rotterdam, Netherlands; 2 ESB Business School, Reutlingen University, Reutlingen, Germany; 3 Faculty of Law and Economics, University of Bayreuth, Bayreuth, Germany; 4 Department of Medical Sciences, Psychiatry, Uppsala University, Uppsala, Sweden; 5 Care Policy and Evaluation Centre, the London School of Economics and Political Science, London, UK; 6 Department of Women’s and Children’s Health, Uppsala University, Uppsala, Sweden; 7 Department of Psychology, Uppsala University, Uppsala, Sweden; 8 Department of Applied Educational Science, Umeå University, Umeå, Sweden

**Keywords:** Diseases, Health care, Signs and symptoms

## Abstract

Peripartum depression (PPD) affects ~12–25% of pregnant and postpartum women worldwide, yet routine screening often fails to capture real-time symptom changes. Digital phenotyping (DP), using data from digital devices such as text entries, sleep tracking, physical activity, social media behavior, and ecological momentary assessments, has been proposed as a complementary approach to support the prediction and early identification for PPD. This systematic review (PROSPERO: CRD42023461325) examined 14 studies published between 2014 and March 2025 that explored passive and active DP data across the antenatal and postnatal periods. Most studies employed observational designs and used the Edinburgh Postnatal Depression Scale as the primary outcome. Passive DP data related to sleep and circadian rhythms were frequently associated with depressive symptoms, whereas findings for physical activity were inconsistent. Active DP data, including language features from text entries, mood logs, semi-random ecological momentary assessments, and social media behavior, were often reported as informative, particularly when combined with personal history or self-reported measures. However, considerable variation across study designs, data sources, analytical approaches, and validation strategies limits direct comparison of findings and prevents causal interpretation. Overall, the evidence remains largely exploratory, and findings should be interpreted cautiously pending more rigorous validation.

## Introduction

Peripartum depression (PPD) affects nearly one in four to one in eight pregnant women^1,2^, adversely influencing parental health, mother-infant interactions^3^, and child development^4^. There is growing interest in incorporating *digital phenotyping* (DP) data, which provide moment-to-moment information (e.g., movement patterns) in natural settings^5^. Systematic reviews and empirical studies have demonstrated that digital phenotyping can validly assess mental states and predict symptom changes in depression and other psychiatric disorders^6–8^, prompting growing interest in its application to peripartum mental health. DP can include *passive measures* (e.g., movement patterns, activity levels^9,10^, heart rate^9^, objective sleep measures^11,12^) and *active self-reports* (e.g., voice recordings, diary entries, open-ended text entries^13–15^, mood logs^15,16^, social media behaviors^17–20^), enabling continuous mental state monitoring^21^, and potentially enhancing early PPD identification and prediction. Most screening methods are restricted to one or two time points and often miss symptom fluctuations during the perinatal period^22,23^.

Accurate prediction, early identification, and monitoring of PPD are essential to personalized care^24,25^. Early screening programs are recommended by major health institutions, including the United States Preventive Services Task Force^26^, the American College of Obstetricians and Gynecologists^27^, the Australian National Guidelines^28^, and the United Kingdom National Institute for Health and Care Excellence^29^. Yet, only 30.8% of women with PPD are diagnosed, and of those, merely 15.8% receive treatment^30^. Furthermore, common screening programs often rely on questionnaires at specific time points, missing many cases.

Conventional self-report screening tools face limitations, including limited sensitivity^31^, symptom disclosure influenced by stigma^32^, and the timing of assessments^33^, resulting in many cases remaining undetected. Although biological factors like hypothalamic-pituitary-adrenal dysregulation and inflammatory markers have been linked with PPD^34^, the absence of consistent biomarkers complicates diagnosis^35^. Biological markers associated with PPD show substantial variability across studies and individuals, limiting their ability to distinguish typical postpartum physiological changes from clinically significant depressive symptoms. Without stable and validated biomarkers, clinicians often rely solely on self-report tools, limiting their ability to capture continuous, real-time changes and early emerging risk patterns. Furthermore, research also indicates that a lack of precise screening tools for early identification or prediction of women at risk for PPD and its potential subtypes^36,37^, exacerbates these challenges.

While psychosocial and environmental risk factors, such as psychiatric history, trauma, poor socioeconomic status, poor social support^38,39^, and vulnerable personality traits^40^, have been identified, integrating DP and mobile health may provide a scalable solution for improving early PPD prediction by capturing complementary behavioral, emotional, and physiological signals that evolve before and after symptom onset^41^. Previous research indicates that multiple digital phenotyping modalities can be applied to detect PPD. Active data, such as mood logs and semi-ecological momentary assessments, contribute by repeatedly sampling self-reported affect and cognitive states, enabling the detection of symptom trajectories and early deviations associated with depressive risk^14,15^. Language-based features derived from open-ended text entries and social media content have also been linked to PPD detection by encoding emotional tone, self-referential processing, and experiential narratives that are difficult to capture using structured questionnaires^13,18^. In addition, passive digital phenotyping data from wearable devices, such as sleep, activity, and physiological measures, have shown associations with depressive symptoms in some studies, although findings vary by modality and timing^9,10^. These findings suggest that integrating complementary digital phenotyping modalities may enhance early PPD prediction, motivating the need for a systematic synthesis of existing evidence.

DP data offers low-cost, high-dimensional, and multi-modal continuous measurements, with potential to refine prediction models and facilitate timely, tailored interventions. However, the impact of different DP types on enhancing prediction accuracy remains unclear. Although digital phenotyping has been systematically reviewed for major depressive disorder and other psychiatric conditions, showing robust associations between smartphone‑derived behavioral features and depressive symptoms^42,43^, to our knowledge, no previous review has synthesized prediction-focused digital phenotyping studies in perinatal populations or compared how different types of DP variables relate to PPD. Recently, DP has shown promise in supporting differential diagnoses^44^, depressive symptom detection^43,45,46^ and PPD symptom prediction^15^. However, the predictive performance varies by time frames^9,14^, and modality combinations^14,47^, and some modalities (such as mobility patterns^48^, step counts^9^, sleep efficiency^11^, content of images posted on social media^17^, heart rate^9^) show no or limited predictive value. This review summarizes available evidence on DP for PPD prediction and identification, including data types, timing, analysis methods, and predictive metrics.

## Results

### Study selection

**Fig. 1:**
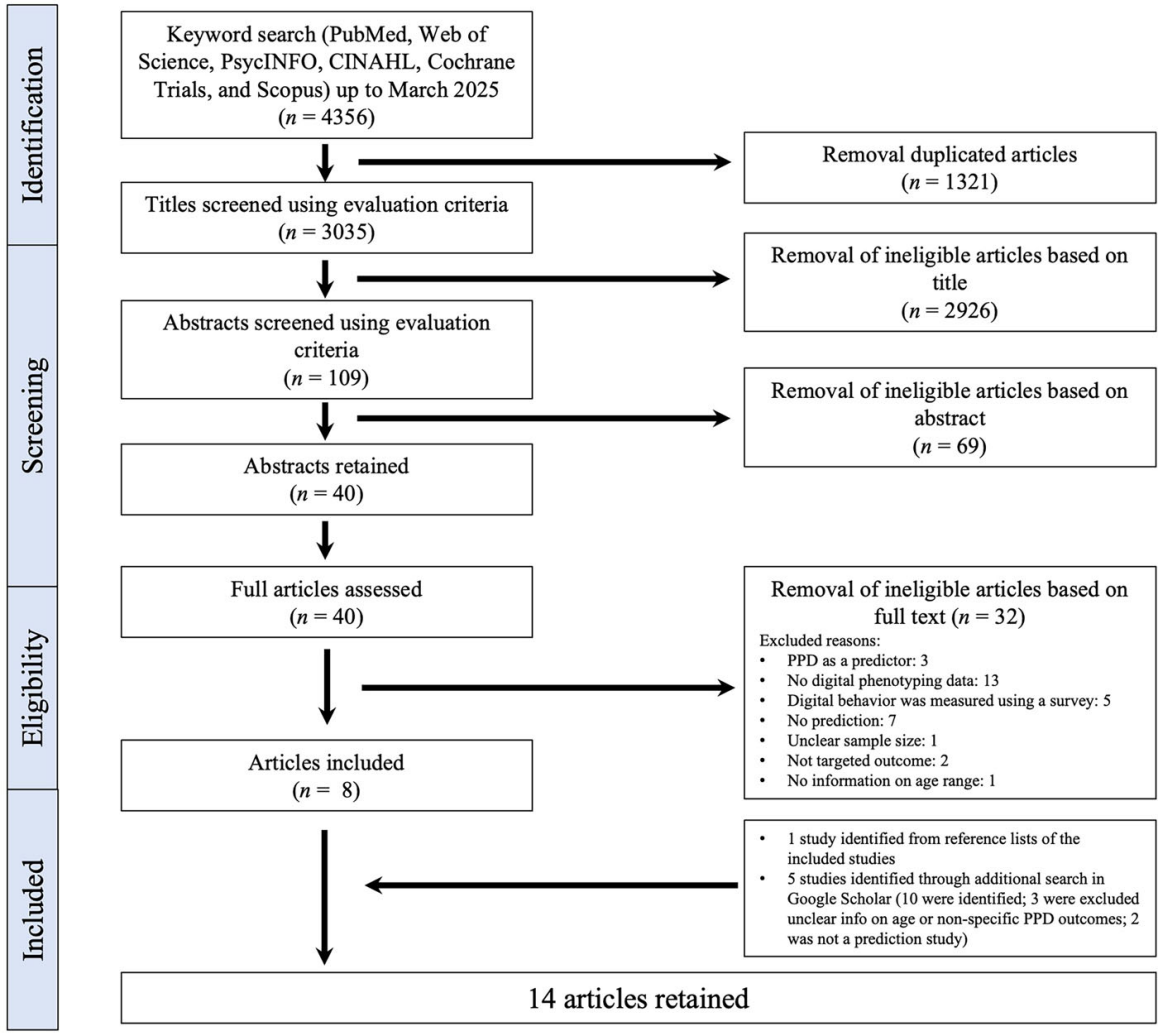
PRISMA flow diagram. This diagram illustrates the study selection process. A total of 4356 records were identified through searches in seven databases up to March 2025. After screening and applying inclusion criteria, 14 studies were included in the final review.

The search process identified 4356 records from databases including PubMed, Web of Science, PsycINFO, CINAHL, Cochrane Trials, and Scopus. After removing 1321 duplicates, 3035 unique records were screened by title, resulting in 109 records for abstract screening. The selection process is illustrated in the PRISMA flowchart (Fig. 1). The final set of included studies reflects the focused scope of this review, which examined the use of digital phenotyping specifically for the prediction and early identification of peripartum depressive symptoms. Studies that primarily addressed other objectives, such as intervention development, feasibility testing of digital adaptations of traditional screening questionnaires, or general monitoring of maternal mental health, were excluded. Applying these criteria resulted in 14 studies that directly investigated predictive models of peripartum depressive symptoms using digital phenotyping data.

### Study characteristics

Table 1 summarizes 14 studies conducted between 2014 to 2025 (March) from the USA (*k* = 8), Sweden (*k* = 3), Canada (*k* = 1), China (*k* = 1), and Germany (*k* = 1). Most employed observational or cross-sectional designs, with sample sizes from 56 to 2062 and participants aged 14 to 45. While the majority were Caucasian, some studies included Asian and African populations. Data sources included social media^12,14,17,18^, outpatient clinics^10,14^, university hospitals^11,15,16,47^, mobile applications^48^, perinatal health care centers^14,49^, and a national research dataset^9,50^. Three studies focused on the antenatal period, three on the postnatal period, and eight included both.

**Table 1:** Summary of study characteristics

First author (year)	Study country	Ethnicity	Sample size	Mean age or age range (years)	Data source	Timing	Maternal health app^a^	Types of digital phenotyping data
Allen^14^	USA	Majority Caucasian (88%)	309	18–45	Outpatient clinics; social media	Antenatal (*n* = 178) and postnatal (*n* = 131)	No	Only active data
Allen^15^	USA	Majority Caucasian (79.5%)	2062	29.5	Patients receiving prenatal care in the university hospital	Antenatal	Yes	Only active data
De Choudhury^18^	USA	Majority Caucasian (77%)	165	30.4^b^	Social media and a parenting website	Antenatal and postnatal	No	Both active and passive data
Fransson^48^	Sweden	Majority Caucasian	1577^e^	32	Mobile application users	Antenatal and postnatal	Yes	Both active and passive data
Hahn^16^	Germany	NA	501	32.2^b^	University hospital	Postnatal	No	Only active data
Hummel^49^	USA	African	572	25^b^	Public antenatal facilities	Antenatal and postnatal	No	Only active data
Hurwitz^9^	USA	NA	Max. 58^c^	Ca. 34.3	National research dataset	Antenatal and postnatal	Yes	Only passive data
Hurwitz^50^	USA	Non- Hispanic White (78.9%)	142	PPD median age = 33.1; non PPD median age = 33.9	National research dataset	Antenatal and postnatal	No	Only passive data
Krishnamurti^13^	USA	Majority Caucasian (79.3%)	1274	29.8	Perinatal healthcare center	Antenatal	Yes	Only active data
Micheletti^12^^d^	USA	Majority Caucasian (59%, including white, Hispanic)	56	31 (18–43)	Obstetrics clinics, support groups, social media	Postnatal	No	Both active and passive data
Pitsillos^11^	Sweden	Caucasian	163	31.8^b^	University hospital	Antenatal and postnatal	No	Only passive data
Slyepchenko^10^	Canada	Caucasian	73	31.27^b^	Obstetrics clinics, midwifery practices, an outpatient psychiatric clinic	Antenatal and postnatal	No	Only passive data
Zhang^17^	China	Asian	419	27.6	Obstetrics clinics	Postnatal	No	Only active data
Zhong^47^	Sweden	Caucasian	915^e^	≥ 18	University hospital	Antenatal	Yes	Only active data

^a^Maternal Health App refers to mobile applications specifically designed for perinatal care, such as pregnancy tracking, mood logging, or communication with healthcare providers.

^b^Studies did not specify the age range in the inclusion criteria or the studies included minors (14 ≤ age < 18).

^c^Due to ethical concerns, this study was not able to provide the exact number in the PPD group because it was less than 20 people. For statistical synthesis, we estimated the max. number of the sample, which was 19.

^d^This study was a master's thesis.

^e^These two studies are based on the same cohort (Mom2B).

### Types of DP data and their predictive results for PPD

**Fig. 2:**
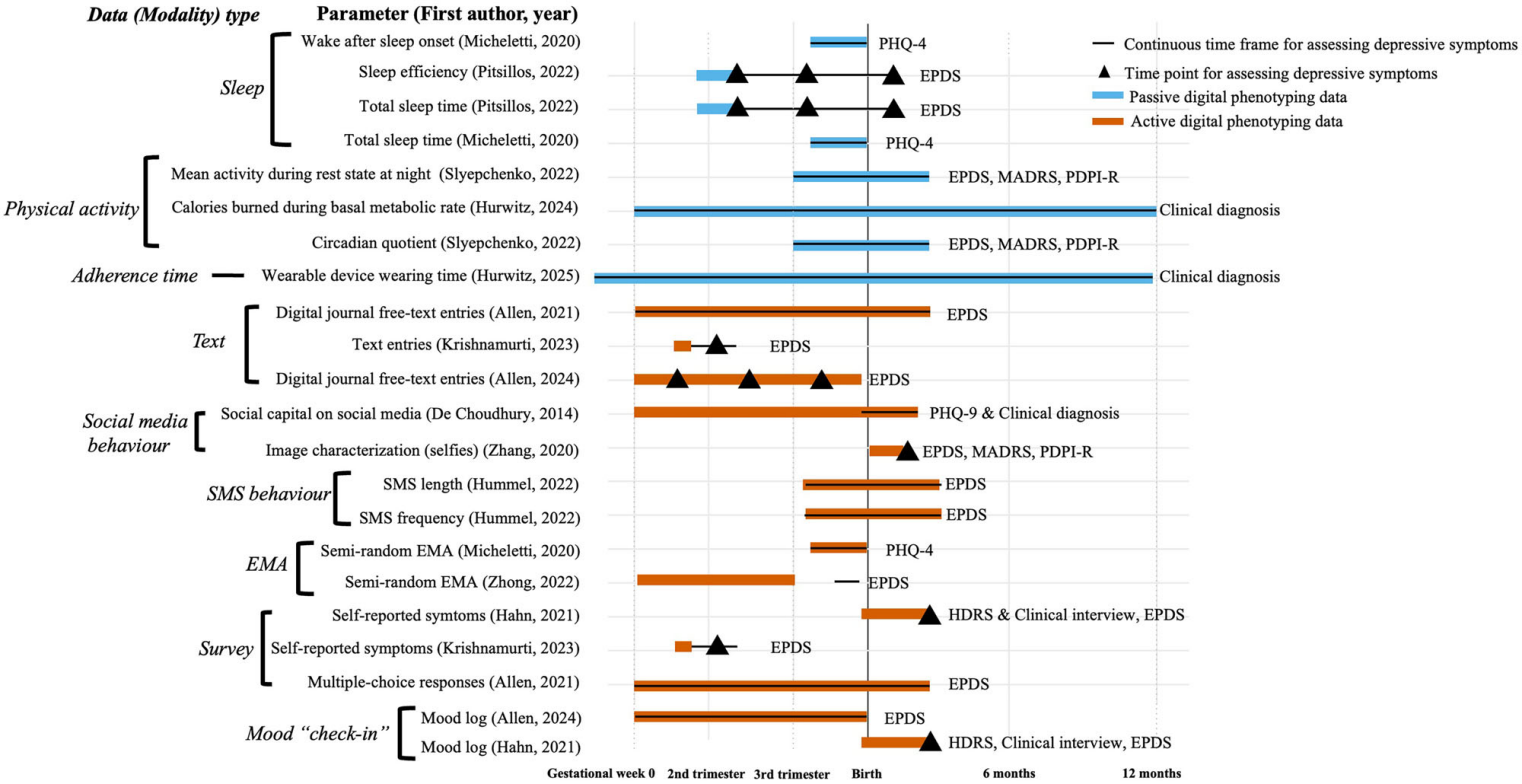
Key timeframes and digital phenotyping data types across studies. Illustration of the included studies, categorized by different digital phenotyping data (modality) types and measurement timing. EMA Ecological Momentary Assessment, EPDS Edinburgh Postnatal Depression Scale, HDRS Hamilton Depression Rating Scale, MADRS Montgomery-Asberg Depression Rating Scale, PDPI-R Postpartum Depression Predictors Inventory-Revised, PHQ Patient Health Questionnaire, and SMS Short Message Service.

**Fig. 3:**
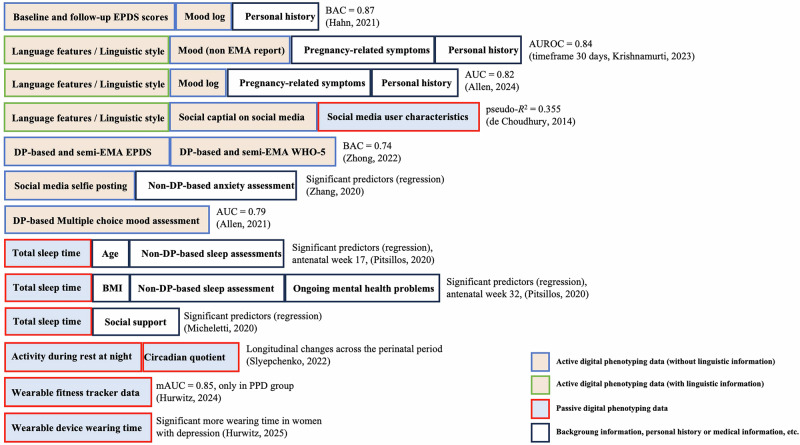
Best modality or combination of modalities across studies. This figure presents the best combinations of data modalities identified across various studies, with each entry represented by the first author's name and publication year. Red-bordered, light blue boxes represent passive digital phenotyping data. Green-bordered, light orange boxes represent active digital phenotyping data with linguistic content, such as open-ended text responses and journals, typically analyzed using natural language models. Blue-bordered, light orange boxes represent active digital phenotyping data without linguistic content. Black-bordered, white boxes represent non-digital phenotyping data, including background characteristics, medical records, personal history, and other conventional clinical information, which are not digital phenotyping data but are included as inputs in the analytical model. The ordering and left-to-right placement of studies and modalities are descriptive and study specific, reflecting the best performing combinations reported by each original study, and do not indicate a ranking of feature importance or predictive strength across studies. AUC Area under the curve, AUROC Area under the receiver operating characteristic curve, BAC Balanced accuracy, BMI Body mass index, DP Digital phenotyping, EMA Ecological momentary assessment, EPDS Edinburgh Postnatal Depression Scale, mAUC Mean area under the curve, PPD Peripartum depression, WHO-5 World Health Organization 5-Item Well-Being Index.

Figure 2 shows key timeframes and measurement types across different studies. Among the 14 studies on PPD, the Edinburgh Postnatal Depression Scale (EPDS) was the primary outcome measure in 9 studies, making it the most commonly used tool. The remaining outcome measures include Patient Health Questionnaire (PHQ-9), Hamilton Depression Rating Scale (HDRS), clinical diagnosis, PHQ-4, clinical interviews, Montgomery-Asberg Depression Rating Scale (MADRS), diagnosis, and Postpartum Depression Predictors Inventory-Revised (PDPI-R). DP modalities included sleep, physical activity, text, social media behavior, SMS behavior, ecological momentary assessment, self-report survey, and mood log. Four studies^10,14,17,49^ primarily utilized DP data for prediction, while other studies incorporated additional data sources into the prediction models, such as sociodemographic data^11,13,16,18^, anamnestic data^16^, electronic health records^9^, self-reported symptoms^13,16,47^, ongoing mental health problems^11^, and self-reported social support^12^. While some DP modalities showed predictive performance, combining multiple modalities generally improved model performance. Due to variability in parameters and methodologies, results could not be statistically synthesized across studies. Figure 3 highlights the top-performing modalities or combinations, and Table 2 details methods and results. Key findings are also summarized in Supplementary Tables 4, 5.

**Table 2:** Summary of methodological details and results across 14 studies

First author (year)	Active digital phenotyping data	Passive digital phenotyping data	Outcome	Statistical techniques	Best digital phenotyping predictor	Validation techniques	Key findings
Allen^14^	Short diary entries, journal text, open-ended and multiple-choice responses	NA	EPDS (≥13), with 9.4% of all women above the threshold	LASSO	Model using the multiple-choice survey	Internal validation: five-fold cross-validation External validation: none	Among 7 sets of language features, only multiple choice reached **AUC of 0.79** in both validation and test models. The combination of all features (e.g. topics, sentiments, syntax, etc.) also reached a good performance in the test model. Moreover, opinion lexicon (positive and negative) and text “taking care of business” in the model built on LDA were top features related to EPDS.
Allen^15^	Survey; routine “check-in”; one-dimensional ecological momentary assessment; open-ended text entries (e.g., journal)	NA	EPDS (≥14) or EPDS > 14 combined self-report of suicidal ideation	Stepwise LR; LASSO; Post-Selection inference for Lasso	Model included personal history, daily mood from “check-in”, and acute pregnancy-related symptoms	A training set (70%), a validation set (15%), and a test set (15%)	Depression models showed varying predictive power (**AUC 0.64–0.83**), influenced by input type. The most accurate models incorporated personal history, daily mood, and acute pregnancy-related symptoms (e.g., severe vomiting, cramping). Daily mood consistently emerged as the strongest predictor of next-month depression. Models that included natural language inputs also tended to improve accuracy, providing richer insights into the lived experience of depression.
De Choudhury^18^	Activities related to social media posts (e.g. active social capital including numbers of updates, uploaded medias, etc.) and the linguistic styles	Passive social capital (comments, wall posts by friends, or likes received)	PHQ-9 with 16.7% women above the threshold	Stepwise LR;	Model combined user characteristics, social capital, content characteristics and linguistic style reached the best pseudo-R^2^	Internal validation: interview on a subset of participants External validation: none	Demographic information explained 14.0% of the variance. Adding user characteristics increased this to 18.3%, social capital to 24.2%, content to 27.9%, and linguistic style to 35.5%. In the final model, the “2nd Personal Pronoun” in linguistic style had the highest coefficient (less frequent use correlated with higher PPD risk), followed by the use of articles and 3rd person pronouns.
Fransson^48^	Survey and audio recording	GPS mobility data (distance from home)	EPDS (≥13)	Linear regression and LR	NA (identification study)	Internal validation: none External validation: none	Mobility patterns from the health app were not significantly related to changes in depressive symptoms.
Hahn^16^	Survey and mood log	NA	HDRS; Clinical interview (p.s. EPDS was considered as a predictor.)	LR	Model including mood log data, baseline and follow-up EPDS scores in the 3^rd^ week postpartum.	Internal validation: strict three-fold cross-validation External validation: external cohort	The authors reported that combining in-clinic and remote self-assessments differentiated women with PPD from healthy women with **AUCs of 0.91** (internal validation) and **0.98** (external validation). For distinguishing PPD from adjustment disorder, AUCs were **0.79** (internal) and **0.88** (external).
Hummel^49^	SMS messaging patterns	NA	EPDS (≥10) with 32.9% of women above the threshold	U-GEE Poisson; M-GEE Poisson	No best digital phenotyping predictors. But “the likelihood of ever sending an SMS” was a highly relevant measure.	Internal validation: none External validation: none	The likelihood of ever sending an SMS was found to vary between mothers experiencing antenatal depressive symptoms and those who did not. Persistent perinatal depression did not demonstrate a significant association with SMS use.
Hurwitz^9^	NA	Heart rate, step counts, activity calories, and sedentary minutes.	Clinical diagnosis	GLM; KNN; RF; SVM	Calories BMR generated by individual RF ML models	Internal validation: three repetitions of ten-fold cross-validation External validation: none	The authors reported that digital biomarkers varied across the pre-pregnancy, pregnancy, and postpartum periods. Based on the ML models, F_*1*_–score during pregnancy (**0.86**) was higher than postpartum (**0.75**) in PPD group.
Hurwitz^50^	NA	Fitbit wearing time	Clinical diagnosis	Correlation	Wearing time	NA	Women with postpartum depression (PPD) showed a trend toward higher Fitbit wear time during the postpartum (72.9% vs. 58.9%, *P* = 0.09) and PPD periods (70.7% vs. 55.6%, *P* = 0.08) compared to those without PPD, suggesting increased adherence possibly linked to hypervigilance.
Krishnamurti^13^	Open-ended text entries; self-report mood score	NA	EPDS (Mild: 7–13; Moderate: 14–19; Severe: 20–30)	LASSO	Language features in a 30-day window (AUC = 0.72)	Internal validation: five-fold cross-validation External validation: none	Language features predicted depression symptoms within a 30-day window (**AUC** **=** **0.72**). Combining these with self-reported current mood improved the predictive model (**AUC** **=** **0.84**).
Micheletti^12^	Semi-random EMA	Objective sleep measures	PHQ-4 (≥3)	Regression	Total sleep time; semi-random EMA (self-report daily social support)	Internal validation: none External validation: none	The authors reported that within-person sleep changes did not predict depressive symptoms. Between-person comparisons showed that mothers with higher depression scores had lower nighttime sleep, higher sleep fragmentation, and infants with more wakefulness after sleep onset. Semi-random EMA data indicated that increased social support significantly reduced negative affect and depression in between- and within-person analyses, respectively.
Pitsillos^11^	NA	Objective sleep measures	EPDS (Antenatal, ≥13; postnatal ≥12)	Multiple linear regression and LR	Total sleep time during pregnancy	Internal validation: none External validation: none	The authors reported that sleep efficiency did not correlate with depressive symptoms during any phase of pregnancy or in the post-partum period. But during pregnancy, women who had the least total sleep time reported higher levels of depressive symptoms, although this association was not observed during the post-partum period.
Slyepchenko^10^	NA	Objective sleep, light exposure, activity level	EPDS; MADRS; PDPI-T	GEE models	Best: longitudinal changes of the mean activity during rest at night; second best: Circadian rhythm strength	Internal validation: none External validation: none	The authors found strong associations between higher circadian rhythm strength and higher mean activity during rest at night with increased depressive symptoms.
Zhang^17^	Content of images on social media (people, object, food, etc.)	NA	EPDS (≥9.5)	LR	NA (correlational results with “selfie posting” as a significant variable in the model)	Internal validation: none External validation: none	Women who posted selfies during the postpartum period were more likely to have postpartum depression.
Zhong^47^	Semi-random ecological momentary assessments	NA	EPDS (≥12), measured between pregnancy week 36 to 42	LR, SVC, KNN, XGBoost, MP	EPDS score week 12 and 24, WHO5 (week 23) based on a multi-modal approach	Internal validation: five-fold cross-validation External validation: none	The authors compared uni-modal and multi-modal models and identified predictive markers related to antenatal depression. With a multi-modal approach the model reaches a **BAC of 0.74**, and an **AUC of 0.80** based on Feature-Level Fusion (XGB) in test set.

*ANN* Artificial Neural Network, *AUC* Area Under the Curve, *AUROC* Area Under the Receiver Operating Characteristic Curve, *BMR* burned during the basal metabolic rate, *EMA* Ecological momentary assessments, *EPDS* Edinburg Postnatal Depressive Scale, *GEE* Generalized estimating equation, *GLM* General Linear Model, *GPS* Global Positioning System, *HDRS* Hamilton Depression Rating Scale, *KNN* k-nearest neighbor, *LASSO* Least Absolute Shrinkage and Selection Operator, *LDA* Latent Dirichlet Allocation, *LR* logistic regression, *MADRS* Montgomery-Asberg Depression Rating Scale, *ML* machine learning, *MP* Multilayer Perceptron, *NA* not applicable, *NB* Naive Bayes, *PDPI-R* the Postpartum Depression Predictors Inventory-Revised, *PHQ* Patient Health Questionnaire, *PPD* peripartum depression, *RF* Random Forest, *RNN* recurrent neural network, *SMS* Short Message Service, *SVM* Support Vector Machine, *U-GEE* Univariable Generalized Estimating Equations with Poisson link, *M-GEE* Multivariable Generalized Estimating Equations with Poisson link, *XGBoost* eXtreme Gradient Boosting Classifier.

### Findings from passive DP data

The included studies reveal three major subgroups of passive digital data (Fig. 2, blue bars). The first focuses on objective sleep measurements^11,12^, highlighting key parameters like sleep patterns and total sleep time. Micheletti et al. monitored sleep continuously over 72 h during the third trimester (31–41 weeks) and found that within-person sleep changes did not predict depressive symptoms assessed six times during that week. However, between-person comparisons indicated that lower nighttime sleep and higher sleep fragmentation were linked to higher depression scores in the third trimester^12^. Similarly, Pitsillos et al.^11^ reported that shorter total sleep time between 11–19 gestational weeks was significantly associated with increased depressive symptoms at 16 and 32 weeks, though not at six weeks postpartum^11^. The second subgroup involved physical activity data, including movement patterns (Global Positioning System, GPS)^48^, heart rate, step counts, activity calories, sedentary minutes^9^, and night activity level^10^. Hurwitz et al.^9^ analyzed Fitbit data from two years pre-pregnancy to two years postpartum and found significant associations between calories burned at resting state and clinical PPD diagnoses at two years postpartum. However, other metrics, such as step count, active minutes, calories burned during physical activity, total distance, and activity type, did not predict PPD^9^. In another study, Slyepchenko et al.^10^ collected physical activity and sleep data at three time points, late pregnancy, 1–3 weeks postpartum, and 6–12 weeks postpartum, and found that nighttime activity and disrupted daily rhythms were robust predictors of PPD symptoms^10^. In contrast, GPS data collected from early pregnancy through one year postpartum showed no significant association with PPD outcomes^48^. Adherence time, the third subgroup capturing wearable device wear duration, showed that women with postpartum depression tended to wear their devices more consistently during the postpartum and peripartum periods. This pattern may reflect behavioral markers such as hypervigilance, which could support early detection and monitoring of mood disorders^50^.

### Findings from active DP data

Six subgroups (Fig. 2) of active DP data were identified: text entries^13,14^, social media behaviors^17,18^, semi-random Ecological Momentary Assessments (EMAs)^12,47^, short message service behavior^49^, survey (self-reported)^13,14,16^, and mood log^15,16^. Text entries were used in two studies. Allen et al.^14^ collected text entries throughout pregnancy and up to 12 weeks postpartum, finding that negative sentiment, physical exhaustion, and lack of positive affect in text were significantly associated with depressive symptoms. The predictive model improved when survey data were included^14^. Similarly, Krishnamurti et al. analyzed text entries and survey data from the first trimester and found that language features, such as sentiment fluctuation, mental health-related language (e.g., “depressed”, “suicidal”, “trauma”), pregnancy-related topics, and the use of first-person plural pronouns (e.g., “we”, “our”, “us”), predicting PPD within a 30-day window^13^. Predictive accuracy increased when combined with self-reported mood data^13^. Social media behaviors were examined in two studies. De Choudhury et al.^18^ monitored social media behavior from early pregnancy to 10 weeks postpartum and found that increased social isolation and reduced social capital (interaction with the contacts on social media) were strong predictors of PPD symptoms^18^. Zhang et al.^17^ observed that mothers who posted more selfies of themselves or their children were more likely to experience depressive symptoms at six weeks postpartum, while other image types (e.g., memes) were not predictive^17^. Short message service (SMS) behavior was assessed by Hummel et al.^49^ who found that fewer and shorter messages during late pregnancy and at 14 weeks postpartum were associated with depressive symptoms. However, persistent depression did not significantly affect the likelihood of sending messages^49^. Two studies used survey data integrated with semi-random EMA for prediction^12,47^. Micheletti et al.^12^ conducted semi-random EMAs six times daily over seven days in late pregnancy, finding that increased social support significantly reduced negative affect and depression in both between- and within-person analyses^12^. Zhong et al.^47^ collected semi-random EMA-based survey data during the first and second trimesters and found that EPDS scores, WHO-5, stress, and anxiety levels were significant predictors of PPD in the third trimester^47^. Self-report surveys were often integrated with other data types to enhance prediction. For example, Hahn et al.^16^ utilized mood logs recorded twice daily from birth to 12 weeks postpartum and found significant associations with PPD outcomes. Combining mood logs with clinical assessments (HDRS, EPDS, and interviews) improved model accuracy and effectively distinguished between women with and without depressive symptoms^16^.

### Statistical approaches

**Fig. 4:**
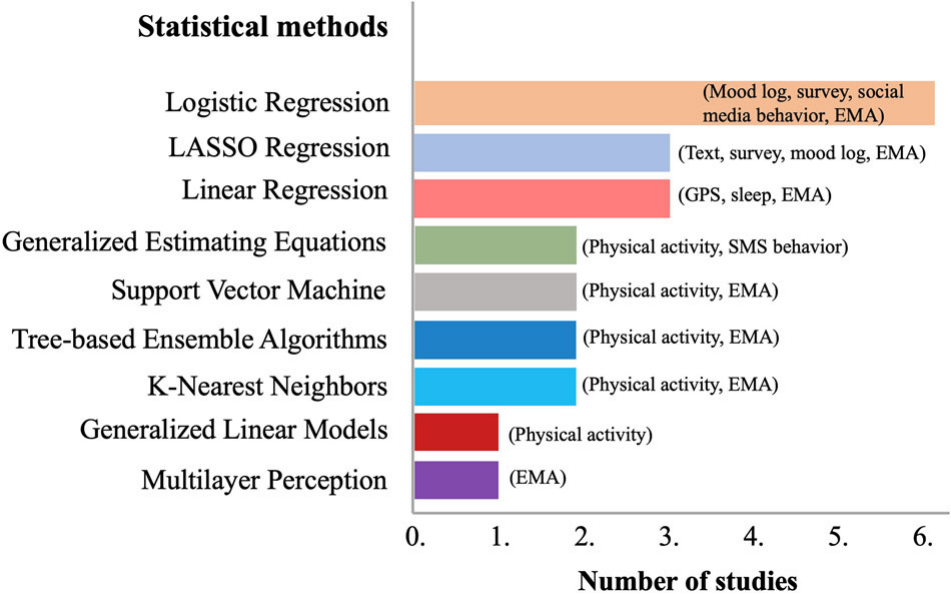
Key statistical methods across studies. Illustration of statistical methods and modalities used in the included studies. The figure presents an overview of the statistical methods applied across the studies. The X-axis indicates the number of studies employing each statistical method, while the Y-axis lists the various methods used. The text content within each bar denotes the different digital phenotyping modalities utilized, rather than their frequency of use. Logistic regression is the most frequently applied method. Abbreviations include LASSO (Least Absolute Shrinkage and Selection Operator), GEE (Generalized Estimating Equations), and XGBoost (eXtreme Gradient Boosting Classifier). Modalities: GPS Global Positioning System, EMA Ecological Momentary Assessment, SMS Short Message Service. For detailed information, please refer to Table 2.

The statistical methods employed across the studies are summarized in Fig. 4 and Table 2.

Traditional statistical approaches, such as Linear Regression, Multiple Linear Regression (MLR), the General Linear Model (GLM), and Generalized Estimating Equations (GEE) were applied in multiple studies (*k* = 6) to structured datasets with predefined assumptions to model the relationships between dependent and independent variables and assess their statistical significance. Linear regression models were applied to analyze independent variables such as GPS data^48^ and sleep data^11,12^. The performance of these models varied significantly based on the inclusion of background information and the specific variables utilized in the analysis. GPS-based distance from home showed no significant link to depressive symptoms, indicating limited predictive value^48^. In contrast, one study found significant associations between total sleep time, wake after sleep onset, and sleep fragmentation during pregnancy and depressive symptoms^12^. Some additional factors contributing to this model were infant sleep hours and social support^12^. Pitsillos et al.^11^ used Multiple Linear Regression and found a significant correlation between total sleep time during pregnancy and depressive symptoms. However, no significant relationship was found with sleep efficiency^11^. Furthermore, Poisson Regression Models were used for count data, with Univariable and Multivariable Generalized Estimating Equations (U-GEE and M-GEE) applied to assess single and multiple predictors, respectively. Specifically, Poisson Regression Models revealed that fewer and shorter SMS messages were sent by women with antenatal and persistent perinatal depressive symptoms compared to healthy women. GEE models demonstrated that higher circadian rhythm strength and increased nighttime activity were significant predictors of depressive symptoms during the perinatal period^10^.

Machine learning (ML) approaches were used in several studies to predict depressive symptoms, employing a range of algorithms tailored to structured and unstructured data types. We divided ML approaches into two categories: (1) algorithms for outcome prediction (e.g., Logistic Regression (LR)^51^, Support Vector Machines (SVM)^52,53^, k-Nearest Neighbors (KNN)^54^, Decision Trees (DT) including Random Forest (RF)^55^ and eXtreme Gradient Boosting (XGBoost)^56^, and Artificial Neural Networks (ANNs), including Recurrent Neural Networks (RNNs)^57^, and simple architectures such as Multilayer Perceptron (MP)^58^; and (2) algorithms for regularization and feature selection, such as the Least Absolute Shrinkage and Selection Operator (LASSO)^59^, which is particularly useful for high-dimensional data with many features relative to the number of observations. Machine learning approaches were used in several studies to predict depressive symptoms, employing various algorithms for data analysis. Logistic Regression was the most common technique (*k* = 6), effectively analyzing data types such as social media behavior^17,18^, mood logs^16^, mobility patterns^48^, and semi-random EMA surveys^47^. For example, stepwise logistic regression accounted for 35.5% of the variance in depressive symptoms by incorporating demographics, user characteristics, content features, social capital, and linguistic styles^18^. Another study found a significant link between posting selfies and peripartum depression, explaining only 20% of the variance^17^. Logistic regression also demonstrated high predictive accuracy in mood logs and surveys, with AUC values of 0.91 and 0.98^16^. The Least Absolute Shrinkage and Selection Operator (LASSO) Regression, used in two studies to analyze language features from text entries, achieved AUCs of 0.72, improving to 0.84 with self-reported mood data^13^, and to 0.79 with multiple-choice surveys combined with free-text responses^14^. Random Forest models were applied in one study to analyze energy expenditure data, achieving AUCs of 0.86 during pregnancy and 0.75 postpartum^9^. However, logistic regression found no significant association between GPS-based distance from home and depressive symptoms^48^.

Multimodal approaches, utilizing various methods like LR, SVM, KNN, MP, and XGBoost, achieved an AUC of 0.83 by integrating diverse longitudinal data^47^, enhancing predictive accuracy. Among unimodal models, XGBoost performed best with an AUC of 0.81^47^. Overall, machine learning techniques exhibited varying efficacy, and the best models depended on data type and structure. Logistic Regression excelled with structured data like mood logs and surveys, while LASSO Regression was more effective for high-dimensional, unstructured data. Additionally, multimodal approaches and Random Forest models for analyzing physiological data demonstrated high effectiveness.

Among the included studies, only one addressed missing data handling^47^ by using most frequent and mean value imputation for categorical and continuous variables, respectively. Regarding model validation, only one out of seven studies conducted both internal and external validation. Hahn et al. analyzed mood logs using Logistic Regression^16^ and reported high predictive accuracy, enhancing its findings’ robustness. In contrast, five studies relied solely on internal validation, analyzing social media behavior^18^, image characterization with Logistic Regression^17^, language features from text entries with LASSO Regression^13,14^, and physical activity data, such as digital biomarkers with Random Forest models^9^.

### Risk of bias and applicability assessment (PROBAST)

**Fig. 5:**
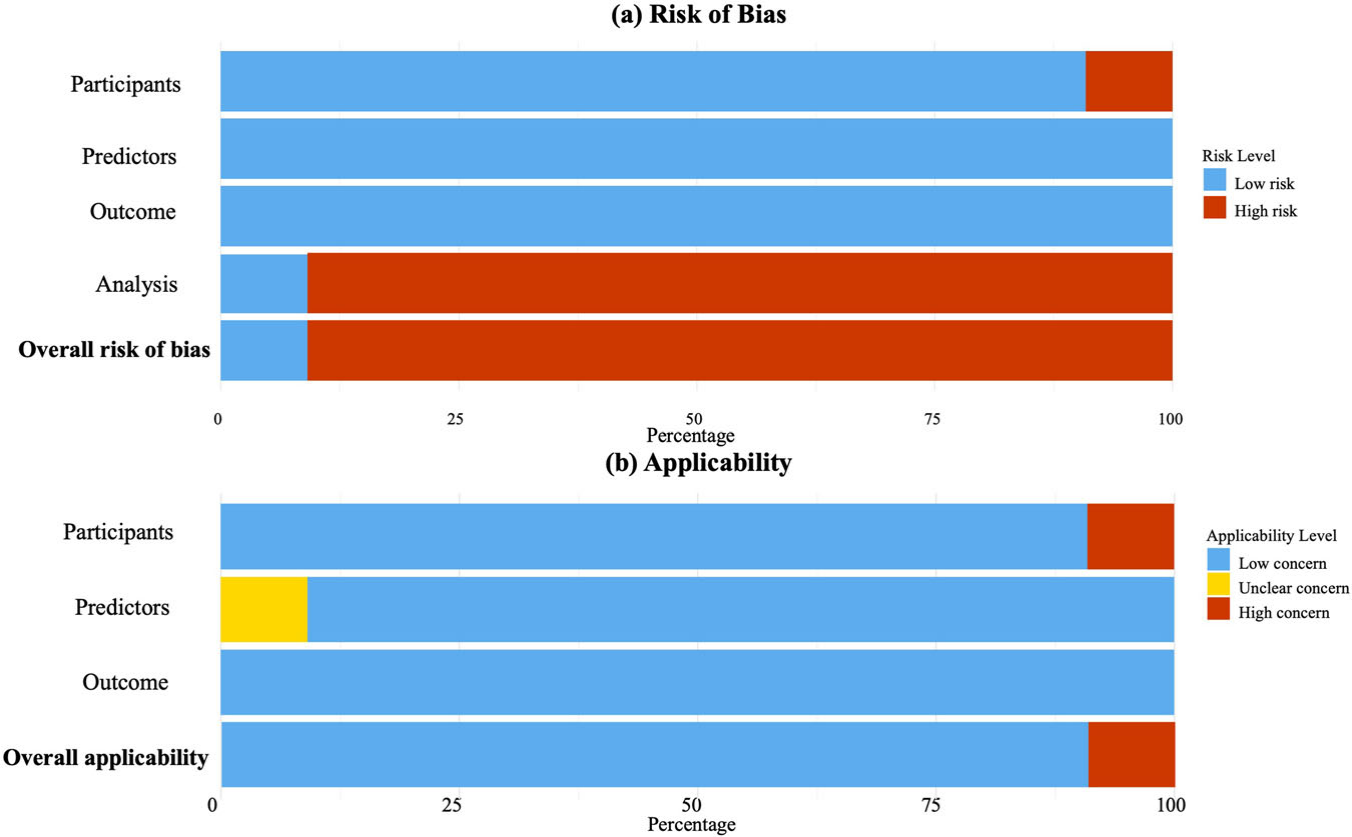
Risk of bias and applicability assessment based on the Prediction model Risk Of Bias ASsessment Tool (PROBAST). Panels **a** and **b** present the risk of bias and applicability assessments, respectively, for the 11 studies that reported predictive modeling. Studies reporting only associations were excluded, as PROBAST is not applicable to those designs.

The details of the PROBAST analysis including 11 studies are listed in Supplementary Table 6 and the results are summarized in Fig. 5. Three studies were excluded from the prediction quality assessment as their study designs focused on association or risk identification rather than the development and validation of formal prediction models, and therefore did not meet the design criteria required for PROBAST evaluation. Most of the studies included in the systematic review were classified as having a high risk of bias overall. Nine studies were rated as high risk in the analysis domain, primarily due to inadequate information on missing data handling and the absence of validation procedures. Additionally, in the participant domain, one study had a group with fewer than 20 participants, and another study failed to report the inclusion and exclusion criteria. For overall applicability, most of the studies exhibited low concern. However, one study was assessed as “unclear” due to the independent variable, and one study raised concerns due to its very small sample size.

## Discussion

This review is the first to identify key DP variables relevant to the early identification and prediction of PPD, summarizing evidence from 14 studies across 5 countries conducted between 2014 and March 2025. While approximately one-third of the studies relied mainly on DP data, most incorporated additional information, such as sociodemographic data, self-reported symptoms, psychological factors, or clinical assessments, into their prediction models. Notably, studies that reported moderate to high predictive performance often leveraged the background and symptom-related information alongside DP features.

Moreover, most existing models remain at a developmental or proof-of-concept phase, contributing to substantial methodological heterogeneity in study design, feature extraction, outcome measurement, analytic strategy, and validation practice. Only a small minority of studies conducted both internal and external validation, and several relied exclusively on internal validation or association-based analyses, limiting the generalizability and clinical interpretability of reported performance. Although several modalities show promise for predicting depressive symptoms, the current literature still reveals a significant gap in translating these findings into large-scale clinical applications. This gap is often driven by challenges in data generalizability, the need for robust internal and external validation, and the complexities of integrating diverse data sources into routine clinical practice^60^. Following this overview, we will discuss some important aspects critical to advancing this field.

Despite the promise of DP for predicting depressive symptoms, it is crucial to acknowledge the risks and biases in data collection and analysis. Several included studies exhibited a high risk of bias, particularly in handling missing data and model validation. While DP methods can generate extensive data, concerns about data quality and missing values can lead to inaccurate measurements and misleading results—issues often overlooked in these studies. For instance, a recent study found that after just three days of inactivity, passive DP data coverage dropped by an average of 19%. Study durations ranged from 4 to 52 weeks, and lower data coverage correlated with less accurate results^61^. Missing values can stem from various factors, including low acceptance of digital collection, device malfunctions, connectivity issues, user disengagement, participant fatigue, and unaddressed software updates. Studies that collect DP data from multiple sources (e.g., GPS, accelerometers, surveys) may encounter challenges in integrating this data^62–65^, which can result in loss or inconsistent recording of information from some modalities. Importantly, identifying these types of missingness and variations across different groups is essential, as different mechanisms of missing data may require different statistical approaches^66^. Moreover, another recent multimodal DP study by Aledavood et al.^67^ combined passive smartphone data with clinical and self-reported measures across psychiatric and healthy control groups and reported an overall passive data missingness of 10.5%, with substantial variation across groups^67^. Taken together, evidence illustrates how clinical status and user engagement can differentially affect data streams and complicate multimodal data integration.

Another issue contributing to a high risk of bias is the lack of model validation. Among the studies reviewed, only Hahn et al.^16^ conducted both internal and external validations. The lack of model validation in both regression and machine learning methods raises critical concerns about the applicability and robustness of predictive models in healthcare. Validation plays a crucial role in regression analysis and machine learning by ensuring the accuracy and reliability of models. In regression-based prediction models, both internal and external validation helps detect overfitting, miscalibration, and uncover biases and inaccuracies in estimating relationships between variables, thereby supporting more reliable clinical interpretations and recommendations^68^. Proper validation against independent datasets ensures that models accurately capture real-world variability, leading to predictions that better reflect patient outcomes across different populations and settings^68,69^. In the context of digital phenotyping for mental health, Birk and Samuel^60^ similarly emphasise that without rigorous validation and attention to data limitations, predictive models risk being biased and clinically misleading^60^.

Although some studies explore similar aspects of DP, there is a lack of consistent terminology, standardized measures, and analytical procedures. For instance, while analyzing active text entries, studies use natural language processing (NLP) or sentiment analysis, but their methods and tools vary significantly, leading to inconsistent results and difficulties in comparison. Emerging evidence suggests that large language models (LLMs) in NLP can predict and monitor adverse mental health outcomes^70,71^. However, the effectiveness of large language models (LLMs) is highly sensitive to hyperparameter tuning, which is crucial for optimizing model performance and ensuring accurate and reliable predictions. Unfortunately, the included studies did not report detailed information on hyperparameters. Properly adjusting these settings is essential to reduce variability and improve the robustness of findings in DP research. Future research should continue to explore innovative statistical approaches while maintaining a focus on interpretability, clinical applicability, and generalizability.

Our review showed that integrating background information, medical history, clinical data, personal history, and real-time, continuous symptom monitoring can improve predictive accuracy. While a comprehensive approach can help capture a richer, more personalized understanding of PPD, realizing this potential requires overcoming several challenges. First, data privacy remains a critical concern, as sensitive personal information must be protected without compromising predictive power^60,72^. Second, interdisciplinary collaboration is vital. For instance, clinicians, data scientists, engineers, and ethicists must work together to create ethically sound, technically robust, and clinically meaningful DP tools^60,73^.

Current evidence is insufficient to identify which DP variables are most predictive of peripartum depressive symptoms for clinical use or to determine optimal measurement timing and duration. While family history and several key biopsychosocial risk factors for PPD have generally been identified as important for prediction efforts^39,74^ in the field, our review suggests that integrating validated assessment tools with semi-random EMA could enhance the ability to capture real-time symptom development, and perhaps identify patterns related to worsening mood. Therefore, conducting a baseline assessment of major psychosocial factors along with passive data collection (e.g., sleep, physical activity) would be valuable. Additionally, ongoing inquiry about emerging risk factors, such as mother-infant interaction patterns, infant-care related behavioral load, and postpartum recovery trajectories captured via digital tools or EMA, is crucial during the peripartum period to ensure comprehensive risk management. These factors often cannot be assessed until after delivery, making it important to maintain open lines of communication with mothers to ensure comprehensive risk management. However, these strategies require robust digital infrastructure, and possibly the use of related wearables, and the addressing of ethical considerations to assist at-risk women effectively. Additionally, caution is needed in integrating DP into clinical practice to avoid exacerbating societal inequalities related to race, gender, and nationality^60^. Future research should focus on the development of a unified framework across disciplines to standardize terminology, features, and methods, incorporating tools like the Clinical Utility Index (CUI)^75^ to enhance decision-making. The integration of insights from fields such as psychology, data science, and clinical medicine will facilitate a more cohesive approach to research and allow for the standardization of practices. This unified framework should be accompanied by comprehensive guidelines for reporting results and implementing open science practices, thereby fostering collaboration and ensuring that findings are both comparable and generalizable across different studies and populations.

In conclusion, digital healthcare applications, particularly those utilizing a multimodal approach, show potential for supporting the prediction for PPD. By integrating medical records, background information, and active DP data such as mood logs or semi-random ecological momentary data, these tools can provide a more comprehensive understanding of mental health. For passive data, so far, sleep parameters seem to be promising. While challenges related to data variability, methodological differences, and bias remain, further research aimed at addressing these issues will be crucial for optimizing the role of digital phenotyping in mental health management.

## Methods

This review was conducted following the Preferred Reporting Items for Systematic Reviews and Meta-Analyses (PRISMA) guidelines (Supplementary Table 1)^76^, and was registered with PROSPERO (CRD42023461325). Six databases, including PubMed, Web of Science, PsycINFO, CINAHL, Cochrane Trials, and Scopus, were searched on the 11th of October 2023 and updated in March 2025. Search results were imported into Rayyan^77^ for duplicate removal and screening. An additional search using Google Scholar was conducted to include more studies before preparing the manuscript.

### Search strategy and eligibility criteria

Two keyword groups were used: for the perinatal period (e.g., “antenatal,” “prenatal,” “postpartum,” “perinatal,” “peripartum,” and “maternal depression”) and another one for DP terms (e.g., “digital phenotyping,” “wearable device,” “active digital data,” “passive digital data,” “smartphone,” “mobile application,” “real-time data,” “ecological momentary assessment,” “text message,” “digital behaviors,” “social media,” and “fitness device”). Boolean logic and search syntax are detailed in Supplementary Table 2. A three-step process (titles, abstracts, full articles) was independently conducted by the first and last authors, retaining articles if either author deemed them relevant. Additional studies were identified via Google Scholar (first 30 pages) and reference checks. Inclusion criteria (based on PICO^78^) covered participants during pregnancy and 12 months postpartum, and studies providing active and/or passive digital information for PPD prediction or identification. Excluded were protocols, reviews, feasibility studies, and those applying digital applications for interventions or using single-time-point questionnaires (Supplementary Table 3).

### Data extraction

For demographic (Table 1), we extracted sample size, ethnicity (when reported), age (reported as mean or age range depending on study reporting), recruitment setting or data sources (online platforms, health apps, etc.), and timing of assessment across the perinatal period. For methodological and analytical details (Table 2), we extracted the types of active or passive data used, PPD assessment tools, outcome definitions and thresholds, statistical or machine learning methods applied, validation strategies (e.g., cross-validation, test set, or external validation), and the best performing model or predictor as defined by the original authors. For quantitative results, we extracted reported performance metrics for machine learning models, including accuracy, area under the curve, sensitivity, specificity, positive and negative predictive values, F1 score, and balanced accuracy, when available (Supplementary Table 4). For non–machine learning studies, we extracted reported effect estimates such as regression coefficients, correlation coefficients, odds ratios, adjusted relative risks, and corresponding statistical significance measures (Supplementary Table 5). When studies reported multiple models or feature sets, results from the best performing model were extracted, with secondary models retained for comparison when explicitly reported.

### Quality assessment

Quality assessment was evaluated using the Prediction model Risk of Bias Assessment Tool (PROBAST)^79^, a structured tool to assess the risk of bias and the applicability of prediction models. Four domains including participants, independent variables, outcomes, and analyses were assessed independently by two authors, and jointly finalized by three authors. Each domain includes several questions addressing potential sources of bias, such as selection of participants, definition of predictors and outcomes, and statistical handling of missing data or overfitting, etc. Evaluators assessed and coded “yes”, “probably yes”, “probably no”, “no”, or “no information,” and assigned a domain-level judgment of low, high, or unclear risk of bias. Applicability concerns were rated in a similar way. In PROBAST terminology, ‘high’ refers to high risk of bias rather than high quality; therefore, a ‘high’ rating indicates methodological concerns rather than strong study quality. Notably, models without external validation were rated high risk unless based on large datasets with internal validation. Studies not reporting prediction results were rated unclear.

## Supplementary information

Supplementary information

## Data Availability

No datasets were generated or deposited during the current study. The data analysed in the review are available in the included studies.
