# Supplementary material for: A review of the application of digital phenotyping in predicting peripartum depressive symptoms
Source: NPJ Digit Med. 2026 Apr 24;9:335. doi: 10.1038/s41746-026-02653-y (PMC13109372; doi:10.1038/s41746-026-02653-y)
Supplement: Supplementary file 1 — Supplementary information [file 41746_2026_2653_MOESM1_ESM.pdf]

Supplementary Table 1. PRISMA 2020 Checklist

Section and Topic	Item #	Checklist item	Location where item is reported
**TITLE**			
Title	1	Identify the report as a systematic review.	Title page
**ABSTRACT**			
Abstract	2	See the PRISMA 2020 for Abstracts checklist.	p. 2
**INTRODUCTION**			
Rationale	3	Describe the rationale for the review in the context of existing knowledge.	p. 3-5
Objectives	4	Provide an explicit statement of the objective(s) or question(s) the review addresses.	p. 5
**METHODS**			
Eligibility criteria	5	Specify the inclusion and exclusion criteria for the review and how studies were grouped for the syntheses.	p. 21-22
Information sources	6	Specify all databases, registers, websites, organisations, reference lists and other sources searched or consulted to identify studies. Specify the date when each source was last searched or consulted.	p. 21
Search strategy	7	Present the full search strategies for all databases, registers and websites, including any filters and limits used.	p. 21 & S. Table 2
Selection process	8	Specify the methods used to decide whether a study met the inclusion criteria of the review, including how many reviewers screened each record and each report retrieved, whether they worked independently, and if applicable, details of automation tools used in the process.	p. 21-22 & S. Table 3
Data collection process	9	Specify the methods used to collect data from reports, including how many reviewers collected data from each report, whether they worked independently, any processes for obtaining or confirming data from study investigators, and if applicable, details of automation tools used in the process.	p. 22
Data items	10a	List and define all outcomes for which data were sought. Specify whether all results that were compatible with each outcome domain in each study were sought (e.g. for all measures, time points, analyses), and if not, the methods used to decide which results to collect.	p. 22 .S. Table 4 & 5
	10b	List and define all other variables for which data were sought (e.g. participant and intervention characteristics, funding sources). Describe any assumptions made about any missing or unclear information.	NA
Study risk of bias assessment	11	Specify the methods used to assess risk of bias in the included studies, including details of the tool(s) used, how many reviewers assessed each study and whether they worked independently, and if applicable, details of automation tools used in the process.	p. 22-23
Effect measures	12	Specify for each outcome the effect measure(s) (e.g. risk ratio, mean difference) used in the synthesis or presentation of results.	p. 22 .S. Table 4 & 5
Synthesis methods	13a	Describe the processes used to decide which studies were eligible for each synthesis (e.g. tabulating the study intervention characteristics and comparing against the planned groups for each synthesis (item #5)).	p. 22
	13b	Describe any methods required to prepare the data for presentation or synthesis, such as handling of missing summary statistics, or data conversions.	NA
	13c	Describe any methods used to tabulate or visually display results of individual studies and syntheses.	NA
	13d	Describe any methods used to synthesize results and provide a rationale for the choice(s). If meta-analysis was performed, describe the model(s), method(s) to identify the presence and extent of statistical heterogeneity, and software package(s) used.	NA
	13e	Describe any methods used to explore possible causes of heterogeneity among study results (e.g. subgroup analysis, meta-regression).	NA
	13f	Describe any sensitivity analyses conducted to assess robustness of the synthesized results.	NA
Reporting bias assessment	14	Describe any methods used to assess risk of bias due to missing results in a synthesis (arising from reporting biases).	p. 22-23
Certainty assessment	15	Describe any methods used to assess certainty (or confidence) in the body of evidence for an outcome.	NA
**RESULTS**			

Section and Topic	Item #	Checklist item	Location where item is reported
Study selection	16a	Describe the results of the search and selection process, from the number of records identified in the search to the number of studies included in the review, ideally using a flow diagram.	p. 6, Fig. 1
	16b	Cite studies that might appear to meet the inclusion criteria, but which were excluded, and explain why they were excluded.	NA
Study characteristics	17	Cite each included study and present its characteristics.	Tables 1
Risk of bias in studies	18	Present assessments of risk of bias for each included study.	p. 15-16, Fig. 4, S Table 5
Results of individual studies	19	For all outcomes, present, for each study: (a) summary statistics for each group (where appropriate) and (b) an effect estimates and its precision (e.g. confidence/credible interval), ideally using structured tables or plots.	Tables 1 & 2<br>Fig. 2 & 3
Results of syntheses	20a	For each synthesis, briefly summarise the characteristics and risk of bias among contributing studies.	p. 7-15
	20b	Present results of all statistical syntheses conducted. If meta-analysis was done, present for each the summary estimate and its precision (e.g. confidence/credible interval) and measures of statistical heterogeneity. If comparing groups, describe the direction of the effect.	NA
	20c	Present results of all investigations of possible causes of heterogeneity among study results.	NA
	20d	Present results of all sensitivity analyses conducted to assess the robustness of the synthesized results.	NA
Reporting biases	21	Present assessments of risk of bias due to missing results (arising from reporting biases) for each synthesis assessed.	Fig. 5, p. 15
Certainty of evidence	22	Present assessments of certainty (or confidence) in the body of evidence for each outcome assessed.	NA
**DISCUSSION**			
Discussion	23a	Provide a general interpretation of the results in the context of other evidence.	p. 16-17
	23b	Discuss any limitations of the evidence included in the review.	p. 20
	23c	Discuss any limitations of the review processes used.	p. 19-20
	23d	Discuss implications of the results for practice, policy, and future research.	p. 19-20
**OTHER INFORMATION**			
Registration and protocol	24a	Provide registration information for the review, including register name and registration number, or state that the review was not registered.	p. 21
	24b	Indicate where the review protocol can be accessed, or state that a protocol was not prepared.	p. 21-22
	24c	Describe and explain any amendments to information provided at registration or in the protocol.	NA
Support	25	Describe sources of financial or non-financial support for the review, and the role of the funders or sponsors in the review.	p. 23
Competing interests	26	Declare any competing interests of review authors.	p. 24
Availability of data, code and other materials	27	Report which of the following are publicly available and where they can be found: template data collection forms; data extracted from included studies; data used for all analyses; analytic code; any other materials used in the review.	NA. Information is all available in supplementary material. No statistical results or codes.

Supplementary Table 2. Databases, keywords, and search strategies used in the review

Six databases, PubMed, PsycINFO, CINAHL, Web of Science, CINAHL, Cochrane Trials, and Scopus, were included in the search. In this document, we listed the keywords and the strategy that were implemented. In addition, Google scholar was used as a supplementary tool.

Keywords

<table>
<tr><th>Keyword Group I</th><th>Keyword Group II</th></tr>
<tr><td>Antenatal depression*
Prenatal depression
Postpartum depression
Perinatal depression
Peripartum depression
Maternal depression</td><td>Digital phenotyping
Wearable device
Passive digital data
Smartphone
Mobile phone
Mobile application
Real-time data
Ecological momentary assessment
Smart phone application
Text message
Digital behavior
Social media
Online social network
Fitness device</td></tr>
<tr><td colspan="2">Search strategy
PubMed search strategy: “(antenatal depression OR prenatal depression OR postpartum depression OR peripartum depression OR peripartum depression OR maternal depression) AND (digital phenotyping OR wearable device OR passive digital data OR smartphone OR mobile phone OR ecological momentary assessment OR smartphone application OR mobile application OR real-time data OR text message OR digital behavior OR social media OR online social network OR fitness device)” ➔
Same keywords and Booleans were applied in other databases.

Antenatal depression* or Prenatal depression* or Postpartum depression* or Perinatal depression* or Peripartum depression* or Maternal depression*

Digital phenotyping* or Wearable device* or Passive digital data* or Smartphone* or Mobile phone* or Mobile application* or Real-time data* or Ecological momentary assessment* or Smart phone application* or Text message* or Digital behavior* or Social media* or Online social network* or Fitness device*

[For Scopus]
Antenatal depression* OR Prenatal depression* OR Postpartum depression* OR Perinatal depression* OR Peripartum depression* OR Maternal depression*

Digital phenotyping* OR Wearable device* OR Passive digital data* OR Smartphone* OR Mobile phone* OR Mobile application* OR Real-time data* OR Ecological momentary assessment* OR Smart phone application* OR Text message* OR Digital behavior* OR Social media* OR Online social network* OR Fitness device*</td></tr>
</table>

Supplementary Table 3. Inclusion–Exclusion criteria for title, abstract, and full article evaluation based on PICO

	Inclusion criteria	Exclusion criteria
**P (population)**	• Women during pregnancy and/or 12 months postpartum	• Women with other psychiatric disorders (e.g., bipolar)<br>• Animal studies
**I (indicator)**	• Women with who provided passive digital information (including mobile phone, wearable fitness devices) and/or active digital data, such as mood log, app text messages, etc.)	• Using digital intervention targeting on women with depression. The intervention program contains modules of psychological treatment, cognitive behavioural therapy, etc.<br>• One-time digital data from questionnaires or surveys (without continuous measurement)
**C (comparator)**	• (If reported) Active digital data, e.g., self-report for monitoring peripartum depression<br>• Studies combined self-report information or electronic health data related to prediction of peripartum depression	• Studies did not apply any digital devices
**O (outcome)**	• Predictive relationships between digital phenotyping patterns and peripartum depression among women during pregnancy and/or 12 months postpartum	• Studies reported only descriptive results or focused on the effect of digital intervention on mothers and/or infants will be excluded.
**Others**	• App or devices for pregnancy monitoring or screening purposes (NOT intervention)<br>• Data content can be: location, activity, sleep, social activity, heart rate variability (HRV), screen time, ecological momentary data, etc.<br>• Screening, random control trials (RCT), and observational studies	• Study protocol / cohort profile<br>• Review<br>• Studies focused on the partner only<br>• Feasibility study testing or only evaluating the acceptance of digital apps<br>• Digital media type (TV, radio or other conventional apparatus)

Supplementary Table 4. Machine learning results reported in 5 included studies

Study	Sample size	Best class /classifier	ACC	AUC	SENS (Recall)	SPEC	PPV (Precision)	NPV	F1 (harmonic mean of Precision and Recall)	BAC
Allen (5-fold C.V.), multiple-choices	247 (pregnant 178, postpartum 131)	LASSO	NA	**0.70**	NA	NA	NA	NA	NA	NA
Allen (Test), multiple-choices	62	LASSO	NA	**0.79**	NA	NA	NA	NA	NA	NA
Allen (5-fold C.V.), all features		[second best] combined all features	NA	**0.66**	NA	NA	NA	NA	NA	NA
Allen (Test), all features		[second best] combined all features	NA	**0.79**	NA	NA	NA	NA	NA	NA
Hahn (Test), mood log combined survey	267	LR	0.87	**0.91**	0.85	0.89	0.45	0.98	0.5884	0.87
Hahn (External validation), mood log combined survey	155	LR	0.935	**0.98**	0.88	0.99	0.88	0.99	0.88	0.93
Hurwitz (Pregnancy, with PPD), calories BMR	max. 19	RF	NA	**NA**	0.89	0.93	0.9	0.92	0.89	0.9
Hurwitz (Postpartum, with PPD), calories BMR	39	RF	NA	**NA**	0.69	0.94	0.75	0.93	0.71	0.81
Hurwitz (Pregnancy, without PPD), calories BMR	max. 19	RF	NA	**NA**	0.85	0.91	0.87	0.91	0.86	0.88
Hurwitz (Postpartum, without PPD), calories BMR	39	RF	NA	**NA**	0.74	0.96	0.76	0.96	0.75	0.85
Krishnamurti (Train + development), natural language, mood and symptoms within 30 days	635	Baseline risk, NLP features, mood + symptoms, 30-day	NA	**0.89**	NA	NA	NA	NA	NA	NA

Krishnamurti (Test), natural language, mood and symptoms within 30 days	122	Baseline risk, NLP features, mood + symptoms, 30-day	NA	**0.84**	NA	NA	NA	NA	NA	NA
Krishnamurti (Train + development), natural language, mood and symptoms within 60 days	635	[second best] Baseline risk, NLP features, mood + symptoms, 60-day	NA	**0.88**						
Krishnamurti (Test), natural language, mood and symptoms within 30 days	122	[second best] Baseline risk, NLP features, mood + symptoms, 60-day	NA	**0.81**						
Zhong (Cross-validation), semi-random EMA	686	XGB, multi-modal model, Feature-level fusion		**0.81**						0.73
Zhong (Test), semi-random EMA	229	XGB, multi-modal model, Feature-level fusion	0.77	**0.80**	0.68	0.79	0.46	0.91	0.79	0.74
Zhong (Cross-validation), psychological health	686	[second best] XGB, uni-modal model		**0.81**						0.74
Zhong (Test), psychological health	229	[second best] XGB, uni-modal model	0.77	**0.81**	0.68	0.79	0.46	0.90	0.78	0.73

Supplementary Table 5. Results reported from none machine learning studies

**Study**	**Modality included in the best model**	**Values**	**Note**
De Choudhury (Social media activities, linguistic styles, and social captial)	Best, 2nd Person pronoun	R square = 0.355, n = 165	
Fransson (GPS and mobility)	No significant finding	r = -0.41, p = 0.27, n = 1577, r(1577) = -0.41	
Hummel (SMS patterns)	Best significant model	Antenatal depression:	Ever vs. never sending an SMS message, n = 572, adjusted relative risk 0.97, p< 0.0001
		Persistent perinatal depression	Likelihood of ever vs. never sending an SMS message, n = 572, adjusted relative risk = 0.96, p = 0.08
Micheletti (Semi-random EMA; Sleep)	[best] self-report, log, social support	n = 56, B = -0.19, t= -5.83, df = 293; r(293) = -0.19	
	[second best]Total sleep time	n = 56, B = -0.11, t= -3.64, df = 247	
Pitsillos (sleep)	[best] Total sleep, 17 weeks antenatal	Multiple log. R. AOR = 3.65, p 0.04, n = 163	
Slyepchenko (Sleep, light exposure, activity level)	[best] mean activity during rest state at night	B = 23.27, p=0.00037, n = 419, r(419) = 23.27	
	[second best] circadian quotien,	B = 6.41, p = 0.027, n = 419	
Zhang (Social media image content)	[best] selfie posting	n = 419, B = 0.82, p = 0.003, odds ratio, 2.27, (CI 1.33-3.87)	

Supplementary Table 6. PROBAST results

First author, year	Risk of Bias				Applicability (concern)			Overall	
	1. Participants	2. IVs	3. Outcome	4. Analysis	1. Participants	2. IVs	3. Outcome	Risk of Bias	Applicability
Allen, 2023	Low	Low	Low	High	Low	Low	Low	High	Low
Allen, 2024	Low	Low	Low	High	Low	Unclear	Low	High	Low
De Choudury, 2014	Low	Low	Low	High	Low	Low	Low	High	Low
Hahn, 2021	Low	Low	Low	High	Low	Low	Low	High	Low
Hummel, 2022	Low	Low	Low	High	Low	Low	Low	High	Low
Hurwitz, 2024	High	Low	Low	High	High	Low	Low	High	High
Krishnamurti, 2023	Low	Low	Low	High	Low	Low	Low	High	Low
Micheletti, 2020	Low	Low	Low	High	Low	Low	Low	High	Low
Pitsillos, 2022	Low	Low	Low	High	Low	Low	Low	High	Low
Slyepchenko, 2022	Low	Low	Low	High	Low	Low	Low	High	Low
Zhong, 2022	Low	Low	Low	Low	Low	Low	Low	Low	Low

IV: the variables used in a prediction model to estimate the risk or probability of an outcome (e.g., postpartum depression).
